# Pangenome diversification and resistance gene characterization in *Salmonella Typhi* prioritized RfaJ as a significant therapeutic marker

**DOI:** 10.1186/s43141-023-00591-w

**Published:** 2023-11-17

**Authors:** Kanwal Khan, Khurshid Jalal, Reaz Uddin

**Affiliations:** 1 Dr. Panjwani Center for Molecular Medicine and Drug Research, International Center for Chemical and Biological Sciences, University of Karachi, Karachi, 75270 Pakistan; 2 HEJ Research Institute of Chemistry International Center for Chemical and Biological Sciences, University of Karachi, Karachi, Pakistan

**Keywords:** Pan-genomic analysis, Drug repurposing, *Salmonella Typhi*, Molecular docking, Dynamic simulations

## Abstract

**Background:**

*Salmonella Typhi* stands as the etiological agent responsible for the onset of human typhoid fever. The pressing demand for innovative therapeutic targets against *S. Typhi* is underscored by the escalating prevalence of this pathogen and the severe nature of its infections. Consequently, this study employs pangenome analysis to scrutinize 119 *S. Typhi*-resistant strains, aiming to identify the most promising therapeutic targets originating from its core genome.

**Results:**

Subtractive genomics was employed to systematically eliminate non-homologous (*n*=1147), essential (*n*=551), drug-like (*n*=80), and pathogenicity-related (*n*=18) proteins from the initial pool of 3351 core genome proteins. Consequently, lipopolysaccharide 1,2-glucosyltransferase RfaJ was designated as the optimal pharmacological target due to its potential versatility. Furthermore, a compendium of 9000 FDA-approved compounds was repurposed for evaluation against the RfaJ drug target, with the specific intent of prioritizing novel, high-potency therapeutic candidates for combating *S. Typhi*. Ultimately, four compounds, namely DB00549 (Zafirlukast), DB15637 (Fluzoparib), DB15688 (Zavegepant), and DB12411 (Bemcentinib), were singled out as potential inhibitors based on the ligand-protein binding affinity (indicated by the lowest anticipated binding energy) and the overall stability of these compounds. Notably, molecular dynamics simulations, conducted over a 50 nanosecond interval, convincingly demonstrated the stability of these compounds in the context of the RfaJ protein.

**Conclusion:**

In summary, the present findings hold significant promise as an initial stride in the broader drug discovery endeavor against *S. Typhi* infections. However, the experimental validation of the identified drug target and drug candidate is further required to increase the effectiveness of the applied methodology.

**Supplementary Information:**

The online version contains supplementary material available at 10.1186/s43141-023-00591-w.

## Background

*Salmonella enterica serovar enterica Typhi* is a flagellated, aerobic, gram-negative rod-shaped bacteria. Typhoid fever, often known as enteric fever, is caused by a strain of *S. Typhi* that is pathogenic only to humans [1]. Ingestion of *S. Typhi* results in a systemic infection because the bacteria enter the mucosal membrane of the gut and spread to other organs such as the liver, spleen, pancreas, and bone marrow [2]. An infection caused by *S. Typhi* has the potential to be fatal since it may affect many body systems at once. Some of the other common symptoms include chills, headaches, fatigue, lack of appetite, a dry cough, a sore throat, and aches and pains in the muscles. These symptoms are comparable to those that are caused by other seasonal viral illnesses. A typical skin symptom is an erythematous maculopapular rash that does not cause itching. This rash most often develops on the chest and belly, although it may also present on the back, arms, legs, and genitalia less frequently [3].

It is estimated that there are 13.5 million cases of typhoid yearly, with 135,000 annual mortalities, and a worldwide incidence of 2.14 per 1000 [4]. Karachi’s active surveillance studies showed 4.7 incidents per 1000 per year, but the exact prevalence in Pakistan is still unknown. Multidrug resistance (MDR) in *S. Typhi* has altered typhoid therapy [5] since it has made first-line medicines (i.e., Amoxycillin, co-trimoxazole, and chloramphenicol) ineffective. Third-generation cephalosporins, especially ceftriaxone, replaced older cephalosporins as the therapy of choice [6]. However, the emergence of extensively drug-resistant (XDR) *S. Typhi* was first reported in 2016 in Karachi, Sindh province [7], that are also resistant to ceftriaxone and fluoroquinolone, and resistance genes for these antibiotics, QnrS and CTX-M15, have been discovered in the genome [6, 8]. This leaves only azithromycin, piperacillin-tazobactam, and carbapenem as potential treatments. According to the National Institute of Health in Pakistan [9], more than 17,000 cases of XDR *S. Typhi* have been documented in the province of Sindh since the first outbreak. Over 20,000 cases of typhoid fever were reported in Pakistan with symptoms similar to COVID-19 during the ongoing SARS-CoV-2 pandemic in June 2020 [10]. The prevalence of bacteria that are resistant to very high doses of antibiotics is increasing, which makes it even more challenging to treat enteric fever globally. To combat this rising resistance, new pharmacological targets, candidates, and effective intervention strategies are urgently needed to combat the *S. Typhi* XDR strain.

Previous work has employed whole genome sequencing (WGS) to investigate the cause of antibiotic resistance and trace the movement of infectious bacteria and viruses [11, 12]. However, wet lab treatments for infections caused by *S. Typhi* multidrug resistance are hampered by the fact that experimental investigation and screening of macromolecules as therapeutic targets are expensive and time-consuming. Comparatively, progress in big data and informatics has significantly cut down on costly and time-consuming traditional laboratory-based experimental techniques [13]. Recent advances in molecular biology and genetics have made feasible pan-genomic research, which examines the whole set of genes across all strains within a clade. This has prompted studies of genetic diversity and is helping scientists pinpoint the kinds of phenotypic variation that exist within any given organism [14]. Since a single-genome sequence cannot capture an organism’s whole genetic repertoire, knowing the complete genome sequence of a strain is essential for understanding its evolution and illness, and it will also enable more specific targets for therapeutic research and drug candidate development. Interest in the pangenome as a tool for combating gram-negative bacteria has increased in recent years such as VRE [15], *Campylobacters* s p[16], and *Shigella flexneri* [17].

To overcome the difficulty of analyzing data from such a diverse set of *S. Typhi* genomes, we constructed a pan-genomic array. Moreover, through the standard drug development process, researchers and scientists work in phases to identify promising new drugs and get regulatory permission for their commercial release. The current drug development process has to be sped up; hence, it is important to look at other methods. Drug repurposing, the practice of finding new therapeutic applications for existing medications, has become more relevant in recent years as it offers a new opportunity to investigate already tested medications for potential new applications

Therefore, the purpose of the present investigation was to examine a large number of *S. Typhi* pathovar isolates (*n*=119) to better understand its pathogenesis and characterize the core and pan-genome subsets to rank the relative importance of their putative drug targets. Our findings give a thorough genetic landscape of the *S. Typhi* species, which is consistent with an earlier study on only a few strains and species. Furthermore, drugs in the FDA’s authorized drug database (*n*=9000) were searched for potential inhibitors of typhoid fever.

## Methods

The present work used pan-genomic analysis for *S. Typhi* resistivity profiling. We evaluated the pangenome and core subsets to find potential therapeutic targets and drug candidates from FDA-approved datasets.

### Data retrieval

The whole genomes of 119 *S. Typhi* strains were obtained from the RefSeq database, available at the National Center for Biotechnology Information (NCBI) [18], which contains the most comprehensive data on the genetic composition of pathogens. The features of these 119 strains, together with their NCBI accession numbers, are reported in Supplementary Table 1. The human proteome was retrieved from the UniProt [19] database. The DrugBank [20] database was used to determine the selected targets’ potential for druggability, while the Database of Essential Genes (DEG) [21] was used to assess the targets’ essentiality. Additionally, to predict the new inhibitors against *S. Typhi*, the FDA-approved library of (*n*=~9000) was screened.

### Pan-Genomics and core genome analysis

The genome variation (i.e., core genome, unique and dispensable genome) observed in the strains of *S. Typhi* was calculated using pangenome analysis *via* BGPA methods [22]. The homologous genes discovered in the pangenome of 119 *S. Typhi* strains were grouped by the USREACH clustering algorithm [23] of the BPGA tool uploading FASTA files as an input with a 70% cutoff value. The pangenome alignment resulted in the discovery of genes found in all strains (i.e., core genome), dispensable genes detected in two or more strains, and unique genes found exclusively in particular strains peculiar to *S. Typhi*. The pangenome and core genome dot plots were constructed by graphing the total number of gene families and genes shared by all strains versus the addition of each genome. The discovered core, accessory, and unique genes were subsequently studied for the resistivity analysis.

Moreover, the whole *S. Typhi* genome was analyzed for antibiotic-resistance genes using data from the Comprehensive Antibiotic Resistance Database (CARD) [24]. Here, we used an automated BLAST alignment against the CARD database, setting the threshold at 70% identity, query coverage, and perfect and strict hits. We also used the maximum likelihood-based UPGMA program to infer the evolutionary relationship between these strains and the core/pan-genome. However, the MUSCLE tool [25] was used with default settings to align these genomes, and BGPA was employed to construct the phylogenetic tree. The Cluster of Orthologous Groups (COG) [26] was used to annotate the genes found in the pangenome for their functions. The number of metabolic pathways in each of these genomes was used to divide the pangenome into three categories: core, accessory, and unique.

### Prediction of drug targets

The predicted core genome of *S. Typhi* was subjected to subtractive genomic analysis for the new and potent therapeutic target discovery. The generated sets of genes from the core genome were submitted to BLASTp [27] against the full human proteome with a threshold *E* value 10^−4^. The human proteome with the highest sequence similarity (>80%) was eliminated. The remaining proteins with no similarities were recovered and subjected to further subtractive genomic analysis.

The non-homolog genes were subsequently explored for essentiality analysis using the Database of Essential Genes [21]. Essential proteins were included in the DEG, and BLASTp was used to analyze them at a threshold of 10^−5^. Sequence-similar proteins to the DEG essential proteins were analyzed further, and the non-essential ones were discarded.

The resulting essential genes of *S. Typhi* were further screened for druggability study. The BLAST of these key genes with an *E* value of 10^−5^ against the full DrugBank datasets [20] of prokaryotes (containing drug targets) was performed to assess the significant drug targets for drug development. The essential proteins with ≥ 30% identity and >50 query coverage against the Drugbank database were extracted as possible therapeutic targets against *S. Typhi*.

Furthermore, pathogenic proteins were studied to identify the genes that produce virulence factors as potential therapeutic targets. Virulence proteins aid bacterial colonization and cellular penetration, ultimately leading to the annihilation of the host immune system. Proteins were categorized according to their virulence using a web-based database called VFDB (virulence factor of pathogenic bacteria) [28]. Proteins from *S. Typhi* were compared to the VFDB using BLASTp with a threshold of 10^−5^.

Novel resistance protein sequences were further predicted from a pathogen’s complete genome and proteome using the antibiotic resistance gene-ANNOTation V6 (ARG-ANNOT V6) software. The FASTA sequence of the remaining candidate proteins was then BLASTed against the resistance proteins in the ARG-ANNOT V6 database at a threshold of 10^−5^.

Subcellular localization was determined for each of the proteins on the final shortlisted proteins using the web-based tools PSORTb version 3.0.2 [29] and Cello v.2.5 [30]. Subcellular localization (SCL BLAST) relies mostly on a BLAST search of the selected proteins against the PSORTb and Cello v.2.5 databases. This includes the extracellular space, the periplasm, the cytoplasm, the cytoplasmic membrane, and the unknown localization proteins. The predicted non-homologous, essential, druggable, and pathogenic proteins were selected as possible therapeutic drug targets for additional structure based and inhibitor assessment analysis.

### Structural modeling and validation

Homology modeling was performed by using a Swiss model server for the final drug target utilizing a fold recognition or threading-based method [31]. The 3D structure of a protein may be built using its FASTA sequence obtained from the NCBI database.

In addition, many bioinformatics programs, including Procheck and PsiPred, were used to verify the accuracy of the determined 3D protein structure. Conformational and topological errors in each residue are evaluated by the Procheck program in the 3D protein structure [32]. Using the high-resolution protein structure that has been experimentally determined and improved, this software establishes a correlation between the provided protein’s several properties and their ideal values. However, the online server Phyre2 was used to predict the secondary structure (i.e., random coils, β-sheets, and α-helices) of the protein.

### Molecular docking and virtual screening studies

Before initiating a docking experiment, it is very necessary to have access to the three-dimensional structure of the protein. A generated 3D model of the protein was selected as a receptor, while a ligand retrieved from a template protein was hypothesized to act as a reference inhibitor. Consequently, docking experiments included a thorough evaluation and optimization of the protein-ligand interaction such as depleting the protein of its ligand and other heteroatoms (including water). For additional processing of proteins, we utilized AutoDock v4.2 [33] such as, all hydrogen atoms were added, non-polar hydrogen atoms were merged, and Kollman charges were added (Morris et al., 2001). Eventually, molecular docking was carried out using AutoDock following the established protocol, i.e., 250 times Lamarckian GA was used with default settings, resulting in 27,000 maximum generations and 2,500,000 evaluations to dock the ligand [6]. The purpose of this re-docking was to validate the applied docking parameters to observe how well it redocked the crystal structure of a bound ligand. For the grid, the X, Y, and Z coordinates were set to 40, 40, and 40 points, and the X, Y, and Z coordinate centers were set at 26.948, 47.209, and 60.47, respectively.

The FDA-approved library of ~9000 compounds was downloaded in SDF dataset format and stored in local bash repositories. The Open Babel tool [34] was used to convert the 2D compound file received into its 3D PDB format. The ligand library’s energy was minimized using the steepest descent iterations (1500) using the MMFF94 force field and the FROG2 software [35]. In addition, gastieger charges were assigned to compounds, and torsion was applied by rotating all rotatable bonds *via* AutoDock. Finally, an optimized compound library was saved in PDBQT format for further virtual screening. The prepared PDBQT library was divided up using the vina_split package of AutoDock vina. The grid box size and spacing used in the redocking experiment were used for the virtual screening. The compounds that fulfilled all criteria were selected for the molecular simulation studies.

### Molecular dynamic simulation studies

The most promising molecule found via virtual screening followed molecular dynamic simulations to assess its stability, flexibility, interactions, and inhibitory potential with the ligand, protein, and protein-ligand complex. The GROMACS v2020 server was used for the molecular dynamics simulations [36], and ligand topology files were generated using the Automated Topology Builder (ATB v3.0) [37] with the gromos54a71 forcefield. The SPC216 solvation model was used within the dodecahedron framework. The margins of the protein box were determined by taking the minimum distance of 1.0 Å from the protein atom to the boundary. Particle Mesh Ewald (PME) was used to hold long-range electrostatic interactions with an 8 Å cutoff. To progressively reduce the restraints, the steepest descent and conjugate gradient techniques were used in sequence for initial minimization. In addition, the system was made more stable once sodium or chlorine ions were introduced to neutralize the overall system. The energy of 50,000 steps of NPT and NVT has been examined as a class ensemble at a temperature of 300 K and a pressure of 1 atm using the Berendsen barostat and a Langevin thermostat algorithm to control the pressure and temperature, respectively. The simulation was eventually conducted for 50 ns with a 50 ps trajectory time interval. In addition, the obtained MD findings were evaluated using the xmgrace program by visualizing RMSFs, RMSDs, Radius of Gyration (Rg), and hydrogen bond analysis.

## Results

### Pan-genome and resistome analysis

**Fig. 1:**
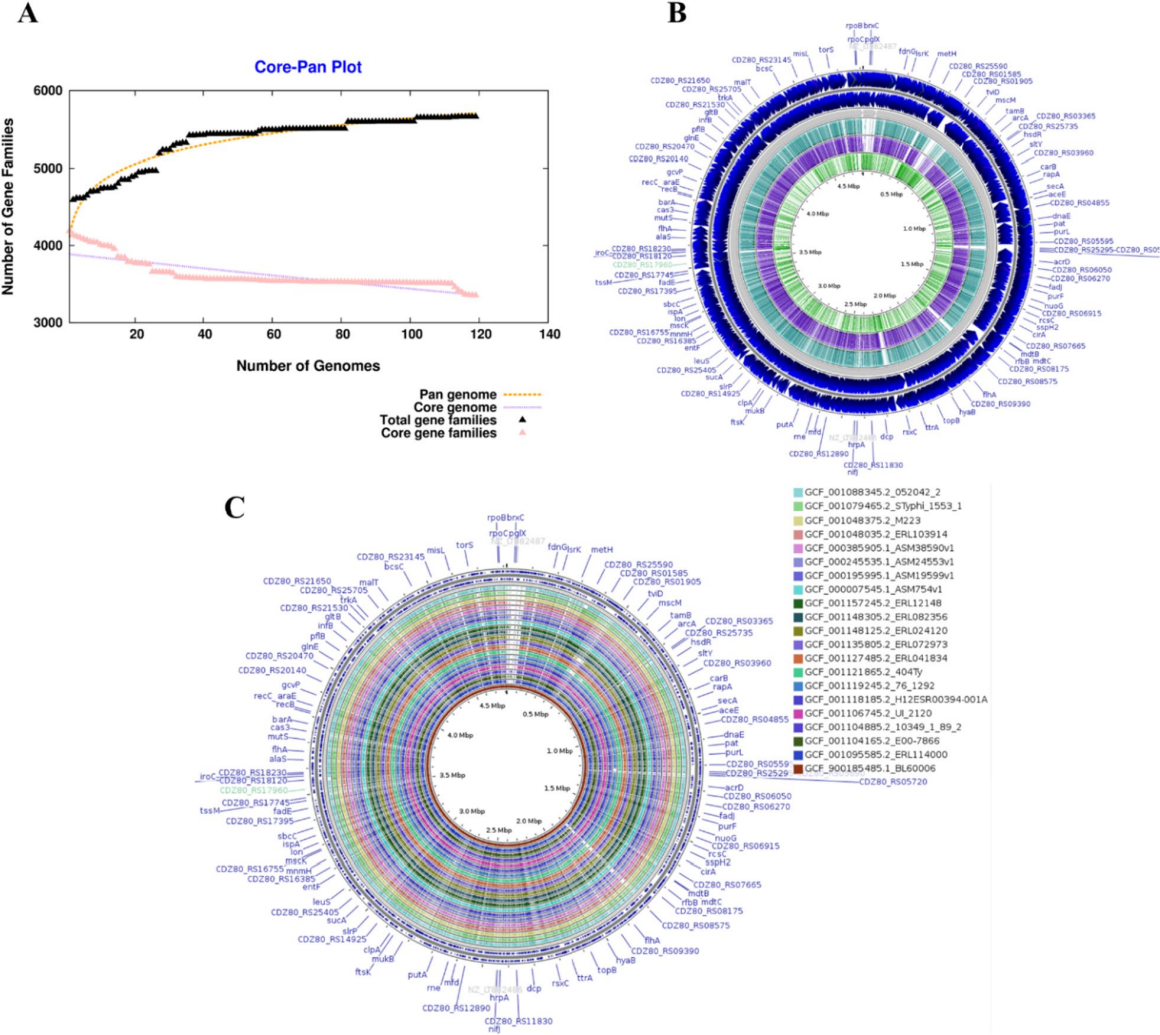
**A** Pan-genome versus core genome dot plot indicating the closeness of 119 *S. Typhi* strains. **B** Alignment of obtained core, accessory, and unique genome of *S. Typhi* pangenome analysis. **C** Pangenome mapping of 20 *S. Typhi* strains from these 119 strains to highlight the alignment taking the latest emerged XDR H58 as a reference

The pan-genome analysis was performed on 119 strains, each of which consists of 5000 CDS sets of genes, to identify a potent drug target against *S. Typhi*. The pangenome analysis identified 3351 conserved genes, or a core genome, across all strains, ~1717 genes, or “accessory genes,” and ~600 strain-specific genes, or unique genes, as shown in Supplementary Table 1. As a result, the pangenome curve of *S. Typhi* represented the Bpan = 0.07 using power-fit value and exponential curve equation through *n* = *a*×*x*^1−*α*^ formula where *n* is estimated pangenome size, *x* is genome used, and *a* is the fitting parameter resulting in almost closed nature of *S. Typhi* (Fig. 1A). Furthermore, the comparative genome study revealed that strain CT18 (GCF_000195995.1) has the highest (i.e., 1165), whereas E98-3139 (GCF_900205295.1) has the least accessory genes (i.e., 653 genes). Furthermore, B/SF/13/03/195 was observed to have 86 absent genes that are exclusively present in other stains while XDR H58 has 42 absent genes, whereas the 80–2002 strain has a maximum of 215 unique genes to all other strains having varied patterns and consisting of 1–87 genes, as shown in Fig. 1B. Figure 1C represents the alignment results for 20 of these 119 strains highlighting the variation of genes.

The resistome analysis of these 119 strains resulted in certain antibiotic resistance genes (ARGs) found in the core, accessory, and unique genomes. The core genome was identified as having 21 resistant genes predicted via strict criteria of resistant gene identifier (RGI). These core resistant genes were involved in antibiotic efflux pump (mdfA, acrA, emrB, msbA, sdiA, baeR, kdpE, CRP, emrR, H-NS, kpnE, and rsmA) resistant to fluoroquinolone, tetracycline, cephalosporin, penam, and monobactams, in antibiotic target alteration (vanG, PmrF, bacA, GlpT, and marR) resistant to the phosphonic acid antibiotic, cephamycin, and glycopeptide antibiotic (Supplementary Table 2), whereas 19 resistant genes were observed in the accessory genome (11 strict and 8 on perfect applied criteria), i.e., CTX-M-15, TEM-1, sul1-2, catI, QnrS1, dfrA, and tetA-B-R, mainly resistant to cephalosporin, monobactam, sulfonamide, phenicol, fluoroquinolone, diaminopyrimidine, and tetracycline *via* antibiotic efflux, antibiotic, inactivation, and antibiotic target replacement pathways (Supplementary Table 3). However, in the unique genome, only 6 resistant genes were identified such as tet(D), SHV-1, APH(3')-Ia, OXA-10, aadA, and cmlA1, resistant to tetracycline, carbapenem, cephalosporin, penam, aminoglycoside, cephalosporin, and phenicol antibiotic *via* antibiotic efflux, and antibiotic inactivation mechanism (Supplementary Table 4).

**Fig. 2:**
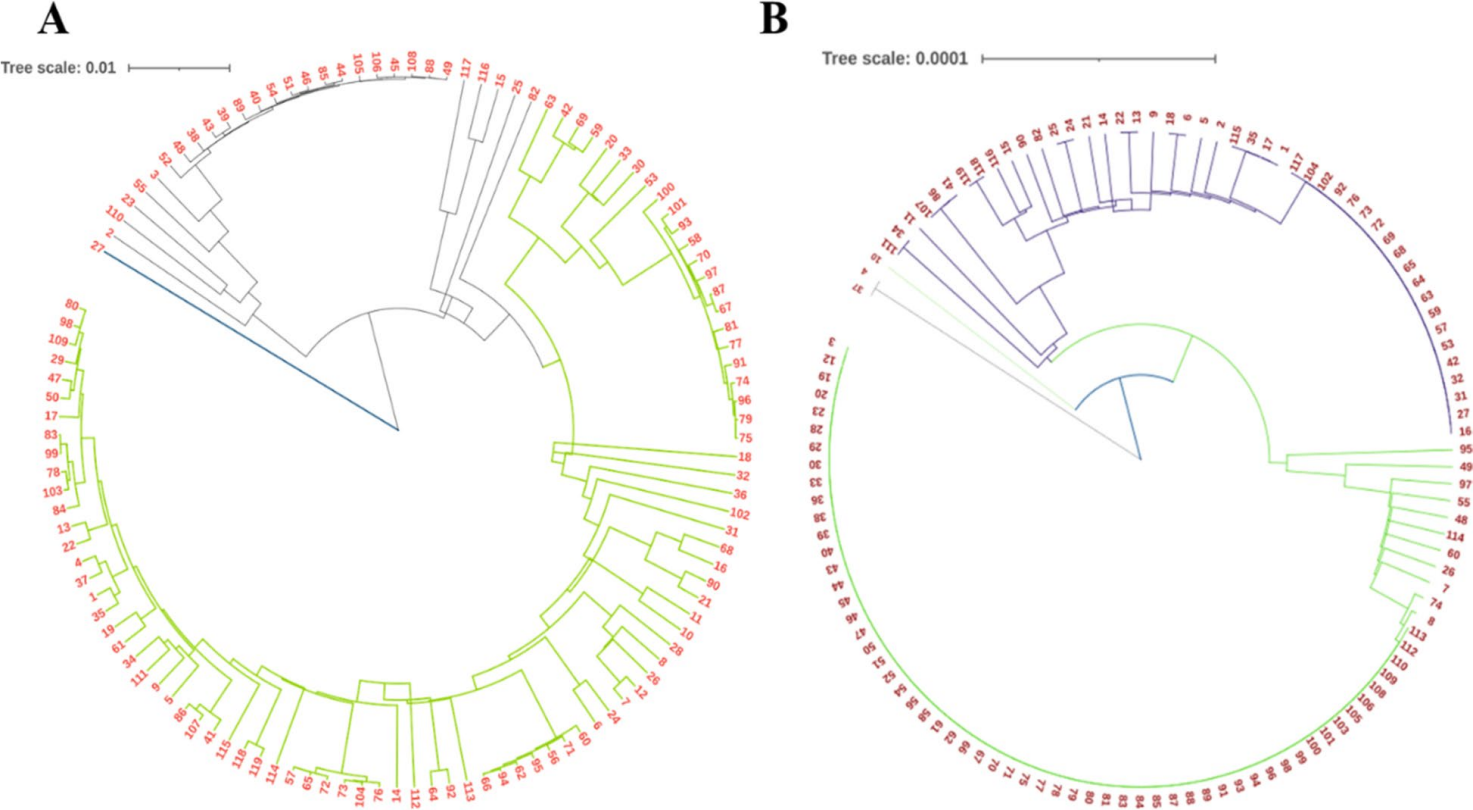
Phylogenomic tree based on the pan-genome (**A**) and core genome (**B**) of 119 *S. Typhi* strains. Each number in the figure represents the serial number of the genome. The detail of these genome number is highlighted in Supplementary Table 1

In addition, the phylogenetic tree constructed from the pan and core genomes demonstrated the strains’ shared ancestry (Fig. 2). Two separate core and pan genomic trees, i.e., generated by core gene alignment and pangenome alignment, were used to investigate the evolutionary connections between these strains. The ratio of pan genes compared strains showed their evolutionary proximity of lineages since more generic strains tend to be found in closer lineages. Phylogenetic analysis showed that all strains in the pan-genome and core genome clustered together were found to be grouped in almost the same clade indicating the similarities.

### Functional enrichment annotation analysis

**Fig. 3:**
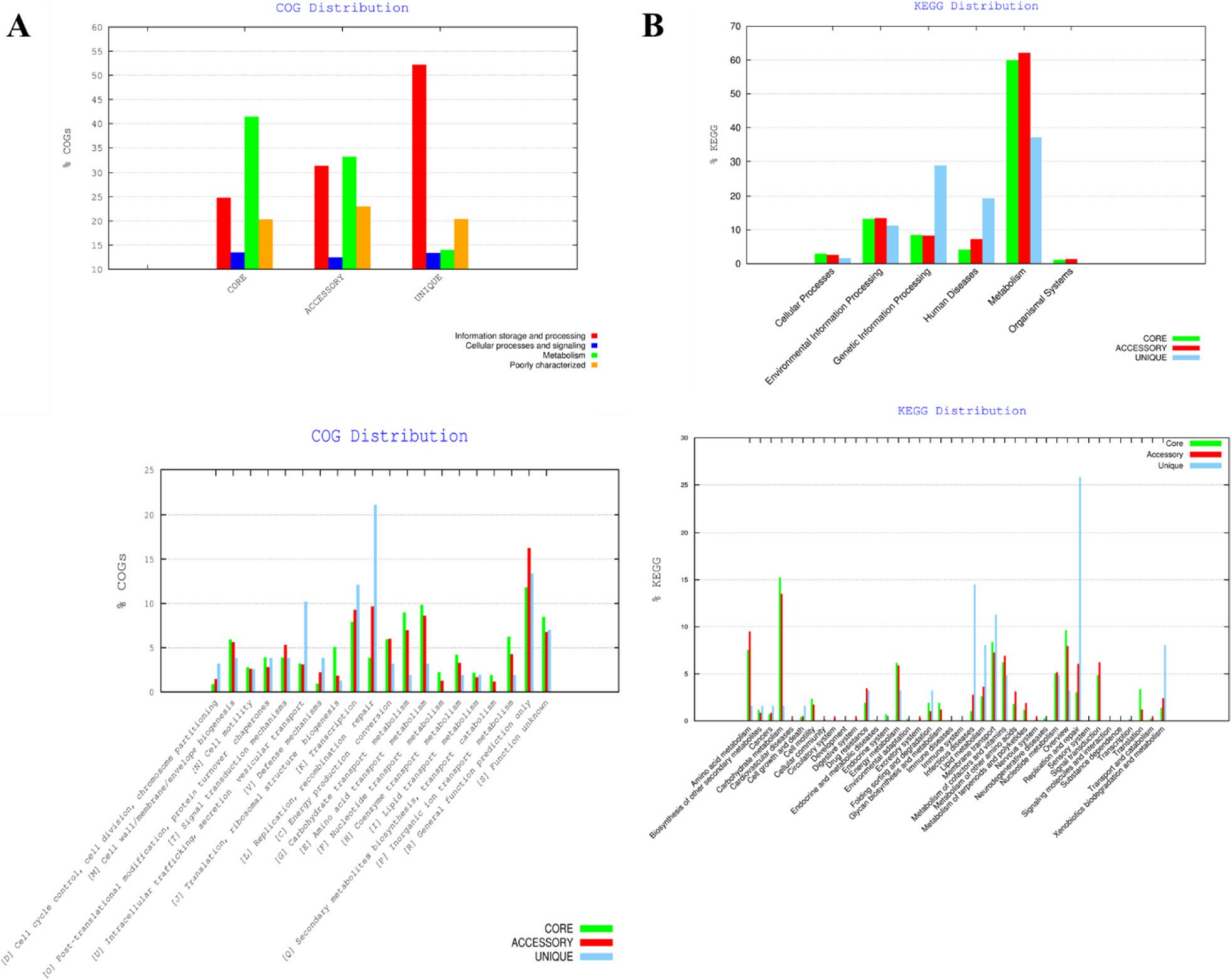
Graphical representation of the gene distribution by metabolic pathway within each core, accessory, and unique genome of the total pan-genome (**A**) via COG analysis (**B**) and KEGG metabolic pathway analysis

The COG functional annotation found that both the core genome and the accessory genome are involved in a variety of metabolic pathway activity, such as information storage and processing pathways. These pathways comprise cell wall, membrane biogenesis, transcription, translation, ribosomal structure biogenesis, inorganic ions, carbohydrates, and amino acid transport metabolism and are mainly involved in poorly characterized pathways (functionally unknown) having functions in general function prediction. However, unique genes were mainly involved in information storage and processing pathways, i.e., replication recombination, repair pathways, transcriptional pathways, cell wall, membrane, and envelop biogenesis, intracellular trafficking, and secretion vesicular transport, respectively (Fig. 3A).

Additionally, the KEGG metabolic pathway classification resulted in the characterization of core, accessory, and unique genomes mainly in environmental information processing, and metabolic pathways validating the predicted COG functional characterization. However, it was observed that a unique genome is primarily enriched in unique genes responsible for the human disease’s conditions and genetic information processing. It was predicted that the involvement of these unique genomes in the genetic makeover of *S. Typhi* is the primary cause of resistant typhoid endemics, thus resulting in the surge of new pathways responsible for human diseases (Fig. 3B).

### Differential proteome mining and therapeutic target identification

Identification of paralogous, essential, pathogenic, and non-homolog proteins is crucial for determining therapeutic targets. Therefore, the drug target identification was processed further using the predicted 3351 core genome of *S. Typhi*.

Over time, homologous proteins have evolved in the bacterial and human cellular systems that are similar. Therefore, treatments intended to bind pathogen target proteins must avoid binding their homologous proteins, which might trigger adverse responses in the host. To identify and select non-homologous proteins among the 3351 core genomes of all 119 *S. Typhi* strains, we performed a BLASTp with a cutoff value of 10^−3^ against the whole human proteome. To reduce the potential for adverse drug reactions, we focused on selecting only non-homologous proteins. Based on their similarities to the human proteome, 1878 proteins were determined to be homologous and so were discarded. For further study, we examined the sequences of the 1473 remaining proteins that were not homologs.

It should be feasible to assess the essentiality of newly submitted proteomes from species now that essential protein data is easily accessible. Essential proteins had a wide range of activities that aided the persistence of infections. Proteins with a high degree of sequence similarity to DEG proteins were prioritized for inclusion in the essential protein set. By comparing these 1473 discovered proteins to the DEG database using BLASTp, 551 proteins were identified as being crucial to the pathogen’s persistent survival. A drug that targets these essential proteins might potentially block *S. Typhi*’s critical activities.

The current study has been improved by evaluating the druggability potential of the prioritized protein sequences. Using the BLAST algorithm on the DrugBank database with an *E* value cutoff of 10^−5^, the druglike potential of 551 essential proteins was calculated. This research led to the identification of 80 proteins in the DrugBank database showing a high degree of homology with FDA-approved drug targets (targets for which experimental evidence exists). The remaining 471 proteins, however, are essential, non-homologous proteins that might be used as therapeutic targets.

In addition, pathogenic proteins play a major role in inflicting infections by releasing virulent factors. The pangenome analysis helped in the identification of such genes, i.e., the shortlisted proteins were BLASTp against the VFDB database. It resulted in the identification of 18 virulent proteins responsible for the pathogenic conditions. Conversely, these 18 proteins were further studied to identify potential therapeutic targets (Table 1).

**Table 1:** Identified 18 drug targets from the core genome of 119 *S. Typhi* along with their drug-like targets and virulent protein features

S. No.	Protein ID	Protein name	Drug	Virulent
1	WP_001125008.1	Outer membrane usher protein	DB04147; DB04233	VFG004229(gi:16759179)
2	WP_000705516.1	Fimbrial outer membrane usher protein StdB	DB04147; DB04233	VFG004286(gi:16761806)
3	WP_001034967.1	TonB-dependent siderophore receptor	DB02415; DB04147	VFG048511 (KOX_13890)
4	WP_000483273.1	Methyl-accepting chemotaxis protein II	DB02365	VFG043040(gi:16765261)
5	WP_000789682.1	Methyl-accepting chemotaxis citrate transducer	DB02365	VFG043209(gb|YP_001006778)
6	WP_000478457.1	Methyl-accepting chemotaxis protein	DB02365	VFG043092(gi:15802298)
7	WP_000094656.1	PAS domain-containing methyl-accepting chemotaxis protein	DB02365	VFG043209(gb|YP_001006778)
8	WP_000533850.1	TolC family outer membrane protein	DB03350	VFG034677(gi:387611073)
9	WP_000824321.1	Porin OmpS2	DB02771	VFG043568(gi:16764916)
10	WP_000865526.1	Porin OmpC	DB02415; DB04233	VFG043568(gi:16764916)
11	WP_000475254.1	Methyl-accepting chemotaxis protein	DB02365	VFG043378(gb|NP_207394)
12	WP_001043666.1	Phosphoporin PhoE	DB04233; DB07084	VFG043568(gi:16764916)
13	WP_000088491.1	Lipopolysaccharide 3-alpha-galactosyltransferase	DB02065; DB02976	VFG013262(gi:148825462)
14	WP_000376863.1	Lipopolysaccharide 1,2-glucosyltransferase RfaJ	DB02065; DB02976	VFG013262(gi:148825462)
15	WP_000174482.1	Lipoprotein insertase outer membrane protein LolB	DB02078	VFG013621(gi:148826132)
16	WP_001024312.1	YceI family protein	DB03232	VFG043388(gi:15645899)
17	WP_000100805.1	DNA starvation/stationary phase protection protein Dps	DB03754	VFG006371(gi:15611298)
18	WP_000434523.1	Thiosulfate sulfurtransferase GlpE	DB02761	VFG044389(gi:6959513)

### Significant and novel drug target prediction

Drugs may reportedly be easily directed toward cytoplasmic proteins, which makes them a promising therapeutic target [38]. Enzymatic proteins are said to be the target of 70% of FDA-approved drugs due to their involvement in numerous distinct pathways. Thus, a single protein, lipopolysaccharide 1,2-glucosyltransferase RfaJ (WP_000376863.1), was shown to be an essential, non-homologous, druggable target against *S. Typhi* out of a total of 18 shortlisted proteins. This protein was selected further for structure-based analysis because of its enzymatic nature, its participation in crucial metabolic pathways, its cytoplasmic localization, and its length (>100 amino acids).

#### Lipopolysaccharide 1,2-glucosyltransferase RfaJ

The enzyme lipopolysaccharide 1,2-glucosyltransferase RfaJ (EC:2.4.1.58) is a key enzyme in the bacterial outer membrane biogenesis; LPS core biosynthesis. It catalyzes the reversible synthesis of UDP-glucose to d-glucosyl, adding the glucose(II) group to the galactose(I) group

of LPS, i.e.,


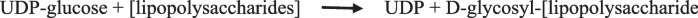


Lipopolysaccharide (LPS) is the most abundant component of the outer leaflet of the gram-negative bacterial outer membrane and is crucial to the membrane’s structural integrity, making it a promising target for the development of novel therapeutics [39]. Han et al. reported that in the combat against the spread of drug-resistant gram-negative bacteria, the lipopolysaccharide biosynthesis pathway has emerged as a promising therapeutic target [40]. Moreover, it is widely studied as a potential drug target against *Escherichia coli* [41]. It is mainly involved in the production of biofilm and is responsible for antibiotic resistance in *Salmonella* serovars [42]. Although it has never been investigated as a possible pharmacological target for *S. Typhi* before, the present work suggests it might be used to combat the resistance shown in XDR strains.

### Protein–protein interaction analysis

Biological processes are regulated by cellular machinery, which is based on protein–protein interactions and their functional annotation [43–45] To fully understand PPI and its significance in the cell, it is necessary to discover numerous interactions and regulate the outcome of these interactions [46]. Based on the STRING data, the shortlisted protein may serve as a hub protein, mediating interactions between other proteins in close proximity to carry out a significant function. Since proteins often operate in groups [44, 45], inhibiting RfaJ’s activity may also disrupt the function of other interactor proteins.

**Fig. 4:**
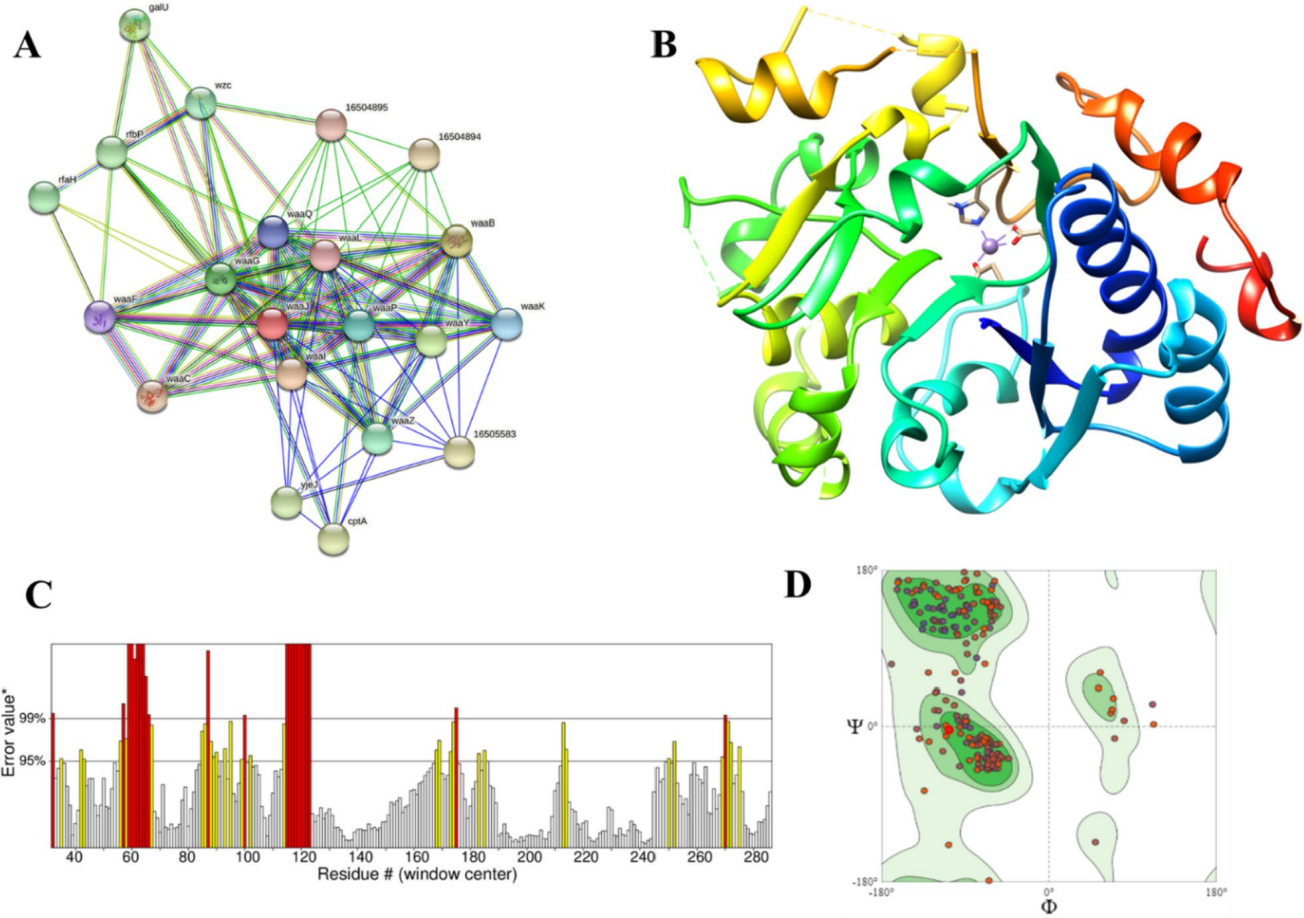
**A** The PPI interaction of RfaJ generated via STRING, **B** modeled structure of RfaJ through the Swiss model, **C** highlights the ERRAT validation of protein, and **D** modeled structure validation through Ramachandran Plot using PROCHECK showing 89.0% residues in the favored region

The STRING database was used to identify waaJ as the protein that facilitates RfaJ’s interactions with other proteins in the vicinity, such as 16504895 (0.642), 16505583 (0.553), cptA (0.519), galU (0.455), rfaH (transcriptional anti-terminator rfah; 0.427), rfbP (0.442), waaB (0.992), waaC (0.595), waaF (0.724), waaG (0.943), waaI (0.993), waaK (0.829), waaL (0.709), waaP (lipopolysaccharide core biosynthesis protein; 0.861), waaQ (0.790), waaY (0.944), waaZ (0.902), wzc (tyrosine-protein kinase etk/wzc; 0.400), and yjeJ (0.505). Isocitrate lyase had 21 nodes, an average node number of 10.7, an average local clustering coefficient of 0.791, a total of 112 edges, a PPI enrichment *p* value of 1.0e16, and 22 predicted edges, according to the PPI data, as shown in Fig. 4A. These proteins play important roles in several processes. The other interactor proteins may similarly cease to function if the RfaJ is blocked. Therefore, it is appropriate to suggest the RfaJ as a prospective therapeutic target.

### Structure prediction and validation

The homology approach of the Swiss Model was used to model the 3D structure of RfaJ. Based on the predicted alignment score, the best-modeled structure was selected, i.e., galactosyltransferase LGTC in complex with UDP-2F-galactose (PDB ID: 1G9R) having 22.78% identity to the RfaJ (Fig. 4B).

For further studies, the modeled structure was validated through ERRAT to predict the quality scores, i.e., 79.215%. Verify3D resulted in a confidence score of 75.67% (Fig. 4C). Moreover, Procheck was used to evaluate the 3D stereometrics of the modeled structure. Comparisons are made between the stereochemical properties of modeled proteins the geometry of their residues and the “ideal” values provided by the Protein Data Bank’s database of highly refined and defined 3D structures of proteins. According to Fig. 4D, the findings of Procheck demonstrate that 88.8% of residues are located in the most preferred areas, while 9.1%, 1.2%, and 0.8% residues lie in extra permitted regions, generously allowed regions, and forbidden regions, respectively.

### Molecular docking and virtual screening studies

Molecular docking is an excellent tool for learning about the interactions between complexes and biological targets. The formed complexes were analyzed with the help of the AutoDock program to get insight into the compounds’ interactions with RfaJ and to determine the probable binding mechanism and energy. RfaJ was used as the template protein’s receptor in a docking study involving URIDINE-5′-DIPHOSPHATE-2-DEOXY-2-FLUOROGALACTOSE (UPF) from the co-crystallized protein as a ligand. UPF was demonstrated to interact with the protein in 250 distinct orientations and conformations. Based on its binding affinity, the ligand was chosen in its conformation 1 state based on its binding affinity, i.e., −14.03 kcal/mol.

**Fig. 5:**
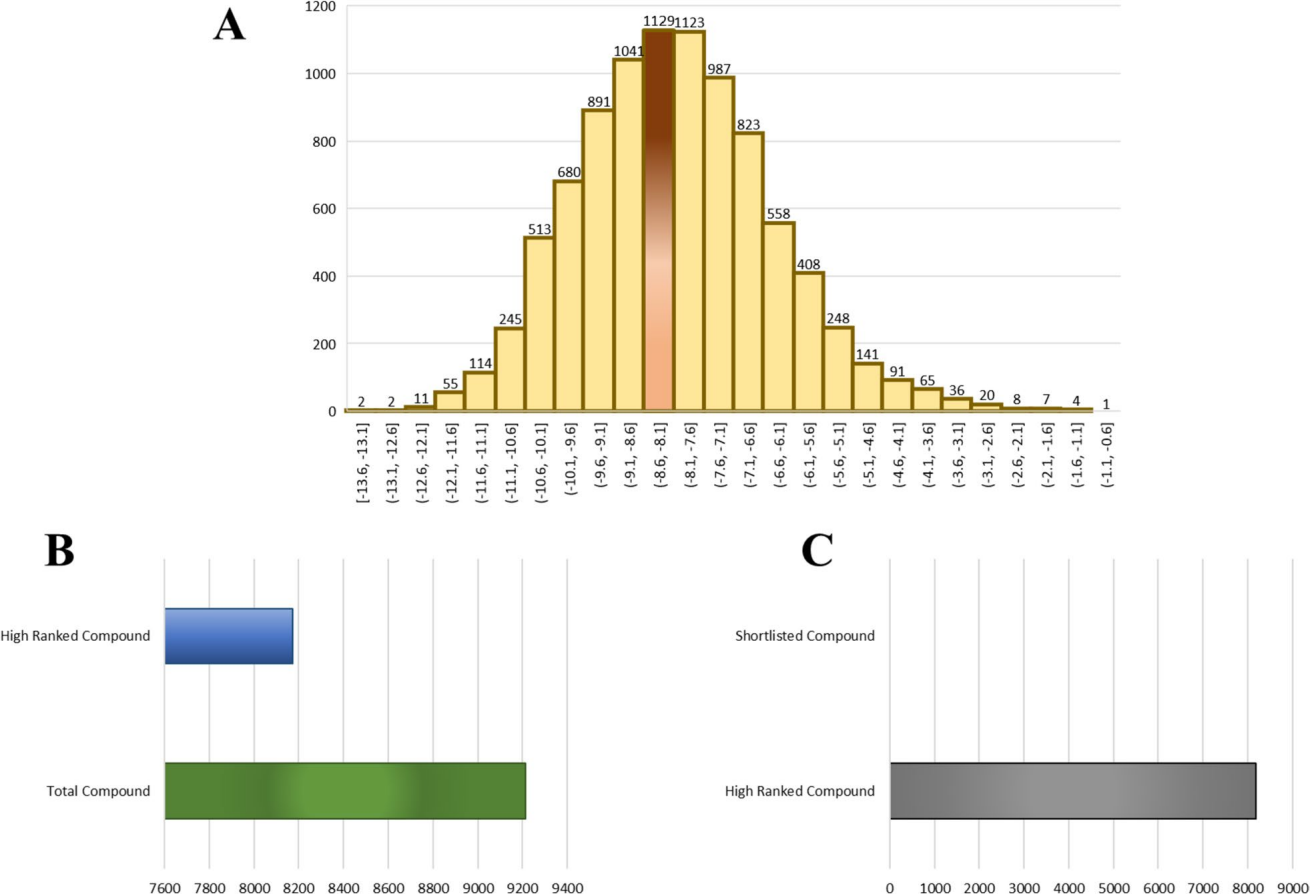
**A** Virtual screening of FDA library against RfaJ, showing the most docked compound at the binding score of −6kcal/mol (brown peak), **B** high docked compounds (having a binding score higher than −6 kcal/mol) compared to the total number of compounds, **C** high docked compound along with the shortlisted four compounds, i.e., DB00549 (Zafirlukast), DB15637 (Fluzoparib), DB15688 (Zavegepant), and DB12411 (Bemcentinib)

Virtual screening employing stringent docking to the active site of RfaJ was performed on the 9213 library, which has been authorized by the FDA. The docking scores were used to produce several docked conformations of the compounds. Hit candidates were filtered out if their binding affinities were less than or equal to −6 kcal/mol. The binding energies varied from −6.0 to −13.6 kcal/mol (Fig. 5A, brown color), for more than 8000 molecules. These compounds were selected for further study because they inhibited RfaJ significantly (Fig. 5B) while having a lower binding affinity. Consequently, only four possible therapeutic candidates, which were DB00549 (Zafirlukast), DB15637 (Fluzoparib), DB15688 (Zavegepant), and DB12411 (Bemcentinib) were selected as they inhibit *S. Typhi* serovar RafJ with high binding potential (Fig. 5C). The detail of these shortlisted compound is provided in Table 2.

**Table 2:**
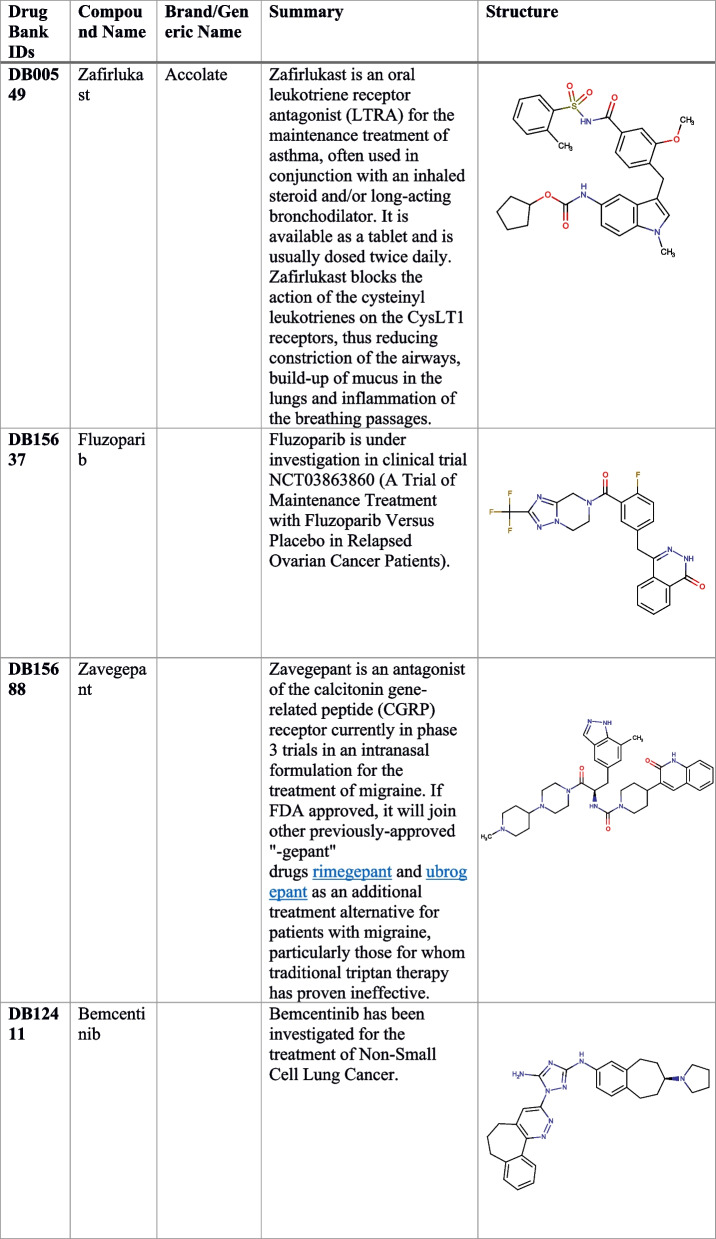
Detail of identified four drug candidates from the FDA library against RfaJ

### Interaction analysis of shortlisted compounds

To comprehend RfaJ pharmacological activity and binding mechanism further, the shortlisted drugs were evaluated utilizing post-molecular docking interaction analysis. In molecular docking, each ligand displayed a variety of interactions with the receptor. The docking rank order based on the docking score is DB12411 > DB15637 > DB15688 > DB00549.

**Fig. 6:**
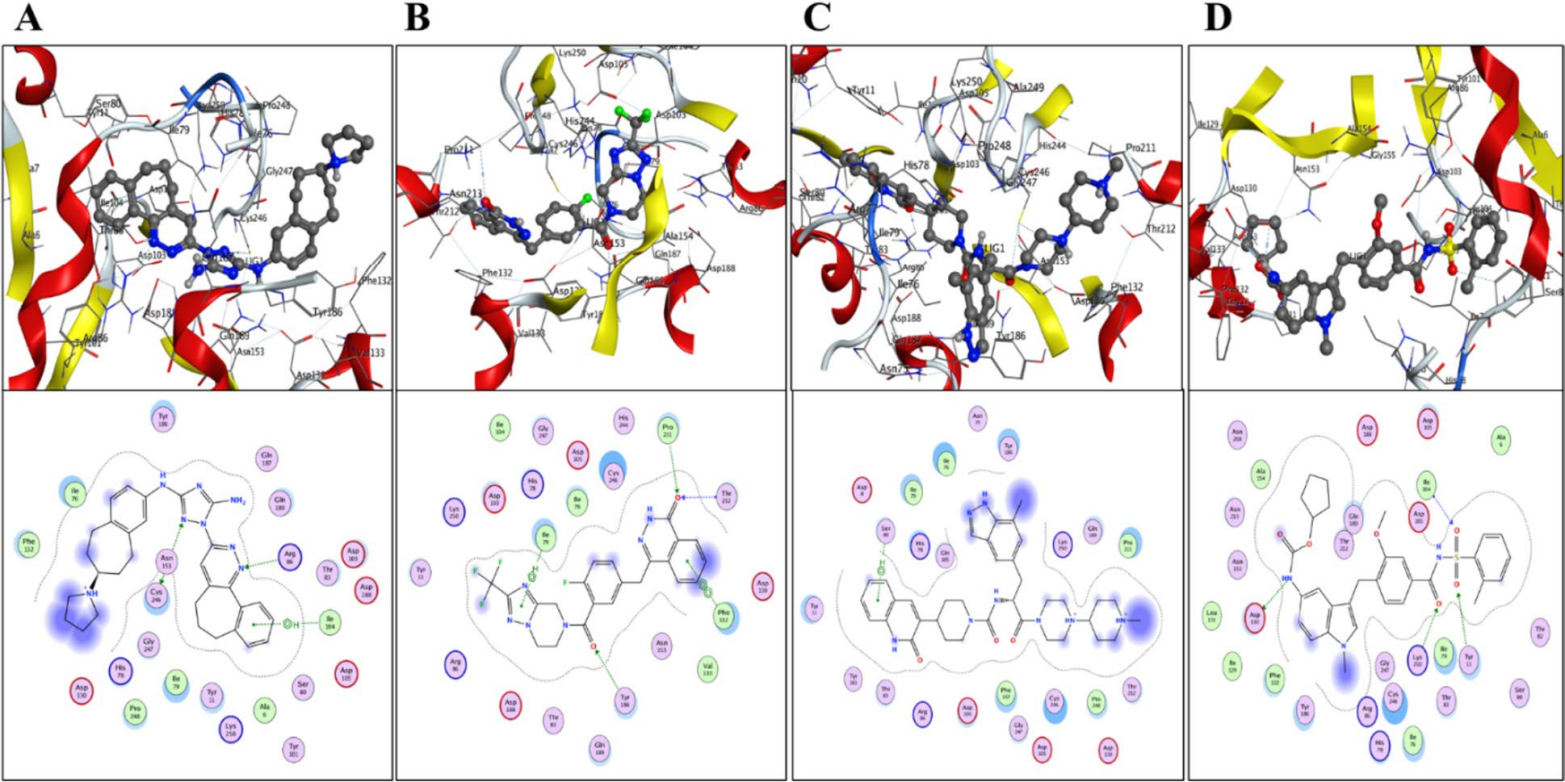
Molecular docking analysis of shortlisted compounds **A** DB15637 (Fluzoparib), **B** DB15688 (Zavegepant), **C** DB12411 (Bemcentinib), and **D** DB00549 (Zafirlukast)

It was observed that DB12411 binds stably within the binding pocket of RfaJ with a binding energy of −13.6 kcal/mol. It mediates three hydrogen bonds with sulfur of Cys246 and nitrogen NH2 of Arg86 with the binding energy of −0.7 and −1.3 kcal/mol having a distance of 3.60 and 2.73Å. Additionally, one pi and a hydrogen bond were observed with Ile104 with a bond distance of 4.59 Å along with an energy of −1.0 kcal/mol correspondingly (Fig. 6A).

The binding energy of DB15637 to the RfaJ binding pocket was −13.2 kcal/mol. Ile79 forms a single aromatic-hydrogen bond with a binding energy of 3.99 kcal/mol and a bond distance of −1.0 through its 5-ring. Phe132 also interacted with the 6-ring through a single pi–pi interaction. As a hydrogen acceptor, it formed four hydrogen bonds with Tyr186, Pro211, and Thr212 through its O18 and O34 (Fig. 6B), with a bond distance of 3.00 and an energy of −0.7 to −2.8 kcal/mol. One aromatic-hydrogen bond between Ser80 and DB15688 was discovered, with a distance of 3.95 and an energy of −1.1 kcal/mol. The resulting binding energy (Fig. 6C) was −12.9 kcal/mol. DB00549 was observed to initiate five hydrogen bonds from Asp130, Lys250, and Tyr11 as a hydrogen donor and acceptor with a distance of 2.73–3.19Å and energies ranging from −0.6 to −3.9 kcal/mol at a binding score of −12.1 kcal/mol (Fig. 6D). A description of binding interactions formed inside the RfaJ active cavity by the selected compounds is shown in Table 3.

**Table 3:** Interaction analysis of four shortlisted compounds in terms of hydrogen bonds and binding scores, distance, and energies

S. No.	Compounds ID	Name	Ligand	Receptor	Interaction	Distance	E (kcal/mol)	Binding scores
1	DB00549	Zafirlukast	N 34	OD1 ASP130	H-donor	3.19	−3.9	−12.1
			N 34	OD2 ASP130	H-donor	3.11	−1.8	
			O 10	NZ LYS250	H-acceptor	2.70	−6.4	
			O 12	N ILE104	H-acceptor	2.87	−2.8	
			O 13	CE2 TYR11	H-acceptor	3.10	−0.6	
2	DB12411	Bemcentinib	N 5	SG CYS246	H-donor	3.60	−0.7	−13.6
			N 5	SG CYS246	H-acceptor	3.60	−0.8	
			N 18	NH2 ARG86	H-acceptor	2.73	−1.3	
			6-ring	N ILE104	pi-H	4.59	−1.0	
3	DB15637	Fluzoparib	O 18	CB TYR86	H-acceptor	3.45	−0.8	−13.2
			O 34	CD PRO11	H-acceptor	3.46	−0.9	
			O 34	N THR212	H-acceptor	3.19	−2.8	
			O 34	OG1 THR212	H-acceptor	3.00	−0.7	
			5-ring	CD1 ILE79	pi-H	3.99	−1.0	
			6-ring	6-ring PHE 132	pi-pi	3.95	−0.0	
4	DB15688	Zavegepant	6-ring	N SER80	pi-H	3.95	−1.1	−12.9

### Molecular dynamic simulation of protein-ligand complex

For the selected inhibitors, molecular dynamic simulations were used to verify the complicated interactions and adaptability. To determine the molecular and atomic motions of the protein-ligand combination at 50ns, the GROMACS server was employed.

**Fig. 7:**
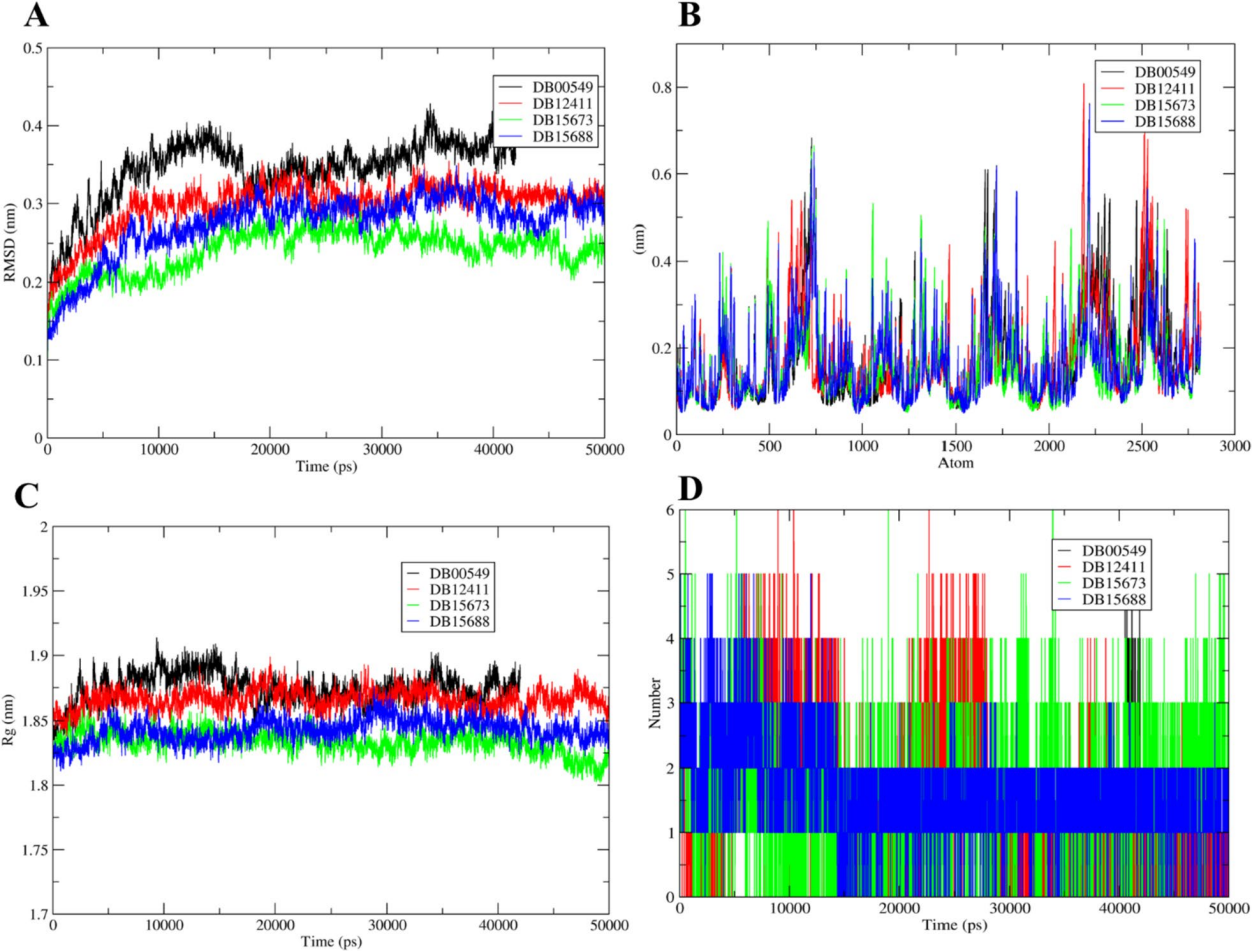
Molecular dynamic simulation results for the shortlisted compound as **A** RMSD, **B** RMSF, **C** radius of gyration, and **D** hydrogen bonds showing DB00549 (Zafirlukast) (black), DB15637 (Fluzoparib) (green), DB15688 (Zavegepant) (blue), and DB12411 (Bemcentinib) (red)

The RMSD analysis for shortlisted compounds showed stability throughout 50 ns simulations within the range of 0.2–0.4 nm resulting in an average RMSD value of 0.3 nm (Fig. 7A). The simulation studies for DB00549 and RfaJ indicate the stability of the complex after 10ns at 3.5–4 nm with mild fluctuations at 11–20 and after 40 ns simulation, whereas DB12411, DB15673, and DB15688 resulted in stability after 5 ns.

The stability of the complex and ligand within the protein’s binding pocket is crucially revealed by the RMSF and Radius of gyration trajectories. A similar pattern of RMSF and gyration was observed for these shortlisted compounds and was observed to be stable within the range of 0.6–0.8 nm (Fig. 7B and C).

The hydrogen bond analysis showed that all the compounds mediate ~5 hydrogen bonds through the 50 ns simulations with mild fluctuation. It was observed that DB00549 mediates 4 hydrogen bonds after 20ns simulations, and DB12411 and DB15637 mediate 5–6 hydrogen bonds with consistence 4 hydrogen bonds throughout the 50 ns simulation, while DB15688 mediates 2–3 persistent hydrogen bonds throughout the 50 ns simulation (Fig. 7D).

## Discussion

Antibiotic resistance poses serious problems for infections caused by gram-negative bacteria, including enteric (typhoid) fever, *Shigella* spp., and pathogenic *Escherichia coli*. Enteric fever is a potentially deadly systemic disease that is spread by *Salmonella enterica serovar Typhi* and other pathovars of *Salmonella* sp. The worldwide spread of the H58 strain has led to the emergence of multidrug-resistant *S. Typhi*, which is resistant to ampicillin, chloramphenicol, and trimethoprim/sulfamethoxazole. Recently, XDR *S. Typhi* strains emerged in Pakistan; resistant to fluoroquinolones and third-generation cephalosporins in addition to the typical MDR phenotype. In South Asian and sub-Saharan African countries, there are an estimated >14 million cases of enteric fever and >135 thousand fatalities annually as a result of poor sanitary conditions. The microbiological, resistance, and evolution processes that lead to the establishment of these resistant strains and the disease of this species have not yet been completely characterized, despite the widespread presence of the XDR strain H58 all over the world.

Given the enormous variety of bacterial genomes, pangenome reconstruction has emerged as a gold standard for deciphering their molecular evolution [47, 48]. There is a lot of variation within species in bacterial genomes due to several causes such as horizontal gene transfer, changes in effective population size, and the persistent colonization of new environments. Pangenome comparisons highlight the evolutionary dynamics of genomes related to important biological processes including speciation, host adaptability, pathogenicity, or the development of antibiotic resistance [49].

Selecting potential therapeutic targets and lead drug candidates against *S. Typhi* is the goal of the current investigation, which is based on the results of a comprehensive pan-genome analysis. The evaluation of 119 antibiotic-resistant genomes indicated the existence of a core genome of 3351 genes shared by all strains and a variable number of distinct genomes and accessory genomes. Furthermore, the comparative genome study revealed that strain CT18 (GCF_000195995.1) has the highest (i.e., 1165) accessory genes while XDR H58 has 42 absent genes. This presence and absence of genes may lead to the activation and deactivation of some new metabolic pathways leading to a resistivity of *S. Typhi*. The number of genes present in these pan-genomes indicates bacterial dynamics and can be utilized to investigate *S. Typhi*’s epidemiological features.

Pan-resistome analysis revealed the presence of antibiotic-resistant genes (ARGs) in all three obtained genomes, i.e., unique, core, and accessory genome. These core resistance genes involving antibiotic efflux pump (mdfA, acrA, emrB, msbA, sdiA, baeR, kdpE, CRP, emrR, H-NS, kpnE, and rsmA) resistant to fluoroquinolone, tetracycline, cephalosporin, penam, and monobactams, and antibiotic target change. Moreover, the reported resistant genes of XDR *S. Typhi* were primarily observed in some of the strains having CTX-M-15, TEM-1, sul1-2, catI, QnrS1, dfrA, and tetA-B-R resistant to cephalosporin, monobactam, sulfonamide, phenicol, fluoroquinolone, diaminopyrimidine, and tetracycline through antibiotic efflux, inactivation, and target replacement routes. While a unique genome was observed to have resistant beta-lactamases such as tet(D), SHV-1, APH(3')-Ia, OXA-10, aadA, and cmlA1 resistant to tetracycline, carbapenem, cephalosporin, penam, aminoglycoside, cephalosporin, and phenicol through antibiotic efflux and inactivation. The obtained results for the resistivity are aligned with the previous results reported [6, 50, 51].

Furthermore, all 119 strains had monophyletic pan and core genomic phylogenetic trees. Since all strains in the pan-genome and core genome belonged to the same evolutionary group, their genome sequences were assessed. These strains may originate from the same environment or the same colony, thus explaining the extremely high degree of similarities in their genomes. Based on the previous study, it is reported that the similarity between sequences can alter the pangenome results (open and close) and the uniqueness of the strains (presence or absence of new genes) [52].

The identified genomes were subjected to functional enrichment analysis and KEGG pathway analysis, both of which revealed a significant overrepresentation of genes involved in metabolic processes such as cell wall, membrane, and envelop biogenesis; secretion, recombination, and replication repair; vesicular transport and transcription; and intracellular trafficking. It has been shown, however, that the unique genome is predominantly enriched in unique genes that are responsible for the circumstances that lead to human illness and the processing of genetic information. It was hypothesized that the participation of these variant genomes in *S. Typhi*’s genetic makeover is the fundamental cause of resistant typhoid endemics, leading to the proliferation of novel pathways responsible for human infections.

Furthermore, a subtractive genome analysis method was used to more effectively decode the core proteome and identify new and effective therapeutic targets. It is one of the most widely applied computational methods for identifying possible therapeutic targets against severe infections. Subtractive genome analysis resulted in the identification of lipopolysaccharide 1,2-glucosyltransferase RfaJ (WP_000376863.1) as one of the potential drug targets. A potential target for the development of new therapies is lipopolysaccharide (LPS), the most prevalent component of the outer leaflet of the membrane of gram-negative bacteria. *Salmonella gallinarum* strains with the RfaJ (WaaJ) and spiC proteins deleted were studied by Zhang et al. They found that these strains exhibited good genetic stability, were less resistant to environmental stresses, and induced antibody production at levels comparable to those seen with the conventional vaccine strain SG9R. Additionally, these outer membrane LPS are classified as pathogenic biomarkers for the detection of UTI caused by *E. coli* [53].

The practice of repurposing pharmaceuticals or finding new uses for existing medications is widely regarded as a cost-effective and time-saving strategy [54]. It goes by several other names: drug rescue, drug repositioning, drug re-profiling, and drug re-tasking. It has been estimated that 75% of currently available medications might be used to treat other conditions [54]. Exorbitant costs, high attrition rates, and long research-to-market clearance times are factors in the current conventional drug development lag. This technique reduces development risk and saves time since the repurposed drug’s safety and pharmacological properties are known. To select potentially effective drug candidates against RfaJ, the present work used UPF as a reference standard and conducted molecular docking and virtual screening of an FDA-approved library. As a consequence, four drugs were selected for further study as possible binders, i.e., DB00549 (Zafirlukast), DB15637 (Fluzoparib), DB15688 (Zavegepant), and DB12411 (Bemcentinib) to inhibit *S. Typhi* serovar. Zavegepant is a small molecule, highly soluble, and calcitonin gene-related peptide (CGRP) receptor antagonist, that has the potential to be an analgesic and immunomodulatory. Bemcentinib, on the other hand, is a selective inhibitor of AXL receptor tyrosine kinase (UFO) that may be taken orally and has the potential to have anti-cancer effects. The small molecule inhibitor of poly-adenosine diphosphate (ADP) ribose polymerase (PARP) 1/2, fluzoparib (SHR-3162), is currently in development for the treatment of BRCA1/2-mutant solid tumors. Zafirlukast inhibits bronchoconstriction by preferentially antagonizing the leukotriene D4 receptor. It has also found new usage in treating oral infections brought on by bacteria like *Porphyromonas gingivalis* and *Streptococcus mutans* [55]. Furthermore, the 50-ns MD simulation study also showed that compounds were stable after 5 ns, with a range of 3.5–4 nm and with slight oscillations. High stability was reported for DB15637 by the simulation studies (RMSD, RMSF, Rg, and hydrogen bond), followed by DB12411, DB15688, and DB00549, respectively.

Finally, the present research pipeline aids in the selection of promising therapeutic targets through high-throughput genome screening and pangenome-resistome analysis. Here, we analyze the genome of *S. Typhi* to acquire vital data for developing future strategies to eradicate this pathogen and define the potential therapies being developed to combat it.

## Conclusion

Pangenome analysis is increasingly being utilized to explore the evolutionary patterns of microorganisms. Although pangenomes can shed light on polymorphic gene content, additional genomic investigations are necessary to definitively ascertain the ecological and adaptive capacities of these species. We analyzed a medically important enteric fever microorganism used in pharmaceutical research. This analysis focused on positive selection, resistance patterns, and recombination landscapes. To achieve this, we constructed a pangenome using genomic data from 119 *S. Typhi* strains. The present investigation utilized a combination of pangenomics and subtractive genomics strategies to identify potential drug targets aimed at combating the 119 *S. Typhi* strain. Consequently, RfaJ, along with other proteins, was considered for its potential in the development of novel pharmaceuticals. Furthermore, a pharmaco-informatics-based repurposing technique was used to screen the FDA-approved library (*n*=9000) for compounds that may inhibit RfaJ. Since drug repurposing requires significantly less time and resources to discover a therapeutic agent than the de novo drug discovery process, it is generally recognized as a highly efficient technique for drug development. As a result, DB00549 (Zafirlukast), DB15637 (Fluzoparib), DB15688 (Zavegepant), and DB12411 (Bemcentinib) were found to be promising inhibitors due to their low estimated binding energy and high stability during ligand-protein interaction. However, experimental validation is necessary to further analyze and enhance the effectiveness of the anticipated targets.

### Supplementary Information

**Additional file 1:** **Table S1.** The detail of 119 strains used for the pangenome analysis.

**Additional file 2:** **Table S2.** Resistance gene identified from the core genome of 119 S. Typhi.

**Additional file 3:** **Table S3.** Resistance gene identified from the Accessory genome of 119 S. Typhi.

**Additional file 4:** **Table S4.** Resistance gene identified from the unique genome of 119 S. Typhi.

## Data Availability

All data generated or analyzed during this study are included in this published article.
