# Supplementary material for: Pangenome diversification and resistance gene characterization in Salmonella Typhi prioritized RfaJ as a significant therapeutic marker
Source: J Genet Eng Biotechnol. 2023 Nov 17;21:125. doi: 10.1186/s43141-023-00591-w (PMC10656401; doi:10.1186/s43141-023-00591-w)
Supplement: Supplementary file 2 — Additional file 2: Table S2. Resistance gene identified from the core genome of 119 S. Typhi. [file 43141_2023_591_MOESM2_ESM.docx]

Supplementary Table 2: Resistance gene identified from the core genome of 119 S. Typhi

| RGI Criteria | ARO Term | SNP | Detection Criteria | AMR Gene Family | Drug Class | Resistance Mechanism | % Identity of Matching Region | % Length of Reference Sequence | |
| --- | --- | --- | --- | --- | --- | --- | --- | --- | --- |
|  |  |  |  |  |  |  |  |  |  |
| Strict | msbA |  | protein homolog model | ATP-binding cassette (ABC) antibiotic efflux pump | nitroimidazole antibiotic | antibiotic efflux | 96.39 | 100.00 |  |
| Strict | emrB |  | protein homolog model | major facilitator superfamily (MFS) antibiotic efflux pump | fluoroquinolone antibiotic | antibiotic efflux | 95.51 | 100.00 |  |
| Strict | Escherichia coli mdfA |  | protein homolog model | major facilitator superfamily (MFS) antibiotic efflux pump | tetracycline antibiotic, disinfecting agents and antiseptics | antibiotic efflux | 87.93 | 100.00 |  |
| Strict | Escherichia coli acrA |  | protein homolog model | resistance-nodulation-cell division (RND) antibiotic efflux pump | fluoroquinolone antibiotic, cephalosporin, glycylcycline, penam, tetracycline antibiotic, rifamycin antibiotic, phenicol antibiotic, disinfecting agents and antiseptics | antibiotic efflux | 91.44 | 100.00 |  |
| Strict | vanG |  | protein homolog model | glycopeptide resistance gene cluster, Van ligase | glycopeptide antibiotic | antibiotic target alteration | 39.44 | 104.30 |  |
| Strict | PmrF |  | protein homolog model | pmr phosphoethanolamine transferase | peptide antibiotic | antibiotic target alteration, antibiotic efflux | 87.74 | 101.55 |  |
| Strict | bacA |  | protein homolog model | undecaprenyl pyrophosphate related proteins | peptide antibiotic | antibiotic target alteration | 97.07 | 100.00 |  |
| Strict | sdiA |  | protein homolog model | resistance-nodulation-cell division (RND) antibiotic efflux pump | fluoroquinolone antibiotic, cephalosporin, glycylcycline, penam, tetracycline antibiotic, rifamycin antibiotic, phenicol antibiotic, disinfecting agents and antiseptics | antibiotic efflux | 98.75 | 100.00 |  |
| Strict | baeR |  | protein homolog model | resistance-nodulation-cell division (RND) antibiotic efflux pump | aminoglycoside antibiotic, aminocoumarin antibiotic | antibiotic efflux | 96.25 | 100.00 |  |
| Strict | kdpE |  | protein homolog model | kdpDE | aminoglycoside antibiotic | antibiotic efflux | 91.96 | 100.00 |  |
| Strict | CRP |  | protein homolog model | resistance-nodulation-cell division (RND) antibiotic efflux pump | macrolide antibiotic, fluoroquinolone antibiotic, penam | antibiotic efflux | 98.57 | 100.00 |  |
| Strict | emrR |  | protein homolog model | major facilitator superfamily (MFS) antibiotic efflux pump | fluoroquinolone antibiotic | antibiotic efflux | 93.14 | 100.00 |  |
| Strict | H-NS |  | protein homolog model | major facilitator superfamily (MFS) antibiotic efflux pump, resistance-nodulation-cell division (RND) antibiotic efflux pump | macrolide antibiotic, fluoroquinolone antibiotic, cephalosporin, cephamycin, penam, tetracycline antibiotic | antibiotic efflux | 94.89 | 100.00 |  |
| Strict | marA |  | protein homolog model | resistance-nodulation-cell division (RND) antibiotic efflux pump, General Bacterial Porin with reduced permeability to beta-lactams | fluoroquinolone antibiotic, monobactam, carbapenem, cephalosporin, glycylcycline, cephamycin, penam, tetracycline antibiotic, rifamycin antibiotic, phenicol antibiotic, penem, disinfecting agents and antiseptics | antibiotic efflux, reduced permeability to antibiotic | 95.24 | 100.00 |  |
| Strict | Klebsiella pneumoniae KpnE |  | protein homolog model | major facilitator superfamily (MFS) antibiotic efflux pump | macrolide antibiotic, aminoglycoside antibiotic, cephalosporin, tetracycline antibiotic, peptide antibiotic, rifamycin antibiotic, disinfecting agents and antiseptics | antibiotic efflux | 76.67 | 100.00 |  |
| Strict | Klebsiella pneumoniae KpnF |  | protein homolog model | major facilitator superfamily (MFS) antibiotic efflux pump | macrolide antibiotic, aminoglycoside antibiotic, cephalosporin, tetracycline antibiotic, peptide antibiotic, rifamycin antibiotic, disinfecting agents and antiseptics | antibiotic efflux | 87.16 | 100.00 |  |
| Strict | rsmA |  | protein homolog model | resistance-nodulation-cell division (RND) antibiotic efflux pump | fluoroquinolone antibiotic, diaminopyrimidine antibiotic, phenicol antibiotic | antibiotic efflux | 85.25 | 100.00 |  |
| Strict | Haemophilus influenzae PBP3 conferring resistance to beta-lactam antibiotics | D350N, S357N | protein variant model | Penicillin-binding protein mutations conferring resistance to beta-lactam antibiotics | cephalosporin, cephamycin, penam | antibiotic target alteration | 51.85 | 96.39 |  |
| Strict | Escherichia coli UhpT with mutation conferring resistance to fosfomycin | E350Q | protein variant model | antibiotic-resistant UhpT | phosphonic acid antibiotic | antibiotic target alteration | 95.03 | 100.00 |  |
| Strict | Escherichia coli GlpT with mutation conferring resistance to fosfomycin | E448K | protein variant model | antibiotic-resistant GlpT | phosphonic acid antibiotic | antibiotic target alteration | 96.24 | 100.00 |  |
| Strict | Escherichia coli AcrAB-TolC with MarR mutations conferring resistance to ciprofloxacin and tetracycline |  | protein overexpression model | resistance-nodulation-cell division (RND) antibiotic efflux pump | fluoroquinolone antibiotic, cephalosporin, glycylcycline, penam, tetracycline antibiotic, rifamycin antibiotic, phenicol antibiotic, disinfecting agents and antiseptics | antibiotic target alteration, antibiotic efflux | 92.36 | 100.0 |  |
