# Supplementary material for: Pangenome diversification and resistance gene characterization in Salmonella Typhi prioritized RfaJ as a significant therapeutic marker
Source: J Genet Eng Biotechnol. 2023 Nov 17;21:125. doi: 10.1186/s43141-023-00591-w (PMC10656401; doi:10.1186/s43141-023-00591-w)
Supplement: Supplementary file 3 — Additional file 3: Table S3. Resistance gene identified from the Accessory genome of 119 S. Typhi. [file 43141_2023_591_MOESM3_ESM.docx]

Supplementary Table 3: Resistance gene identified from the Accessory genome of 119 S. Typhi

| RGI Criteria | ARO Term | SNP | Detection Criteria | AMR Gene Family | Drug Class | Resistance Mechanism | % Identity of Matching Region | % Length of Reference Sequence | |
| --- | --- | --- | --- | --- | --- | --- | --- | --- | --- |
|  |  |  |  |  |  |  |  |  |  |
| Perfect | CTX-M-15 |  | protein homolog model | CTX-M beta-lactamase | cephalosporin, penam | antibiotic inactivation | 100.0 | 100.00 |  |
| Perfect | TEM-1 |  | protein homolog model | TEM beta-lactamase | monobactam, cephalosporin, penam, penem | antibiotic inactivation | 100.0 | 100.00 |  |
| Perfect | sul1 |  | protein homolog model | sulfonamide resistant sul | sulfonamide antibiotic | antibiotic target replacement | 100.0 | 100.00 |  |
| Perfect | catI |  | protein homolog model | chloramphenicol acetyltransferase (CAT) | phenicol antibiotic | antibiotic inactivation | 100.0 | 100.00 |  |
| Perfect | QnrS1 |  | protein homolog model | quinolone resistance protein (qnr) | fluoroquinolone antibiotic | antibiotic target protection | 100.0 | 100.00 |  |
| Perfect | dfrA7 |  | protein homolog model | trimethoprim resistant dihydrofolate reductase dfr | diaminopyrimidine antibiotic | antibiotic target replacement | 100.0 | 100.00 |  |
| Perfect | dfrA14 |  | protein homolog model | trimethoprim resistant dihydrofolate reductase dfr | diaminopyrimidine antibiotic | antibiotic target replacement | 100.0 | 100.00 |  |
| Perfect | qacEdelta1 |  | protein homolog model | major facilitator superfamily (MFS) antibiotic efflux pump | disinfecting agents and antiseptics | antibiotic efflux | 100.0 | 100.00 |  |
| Strict | MdtK |  | protein homolog model | multidrug and toxic compound extrusion (MATE) transporter | fluoroquinolone antibiotic | antibiotic efflux | 99.16 | 102.32 |  |
| Strict | tet(B) |  | protein homolog model | major facilitator superfamily (MFS) antibiotic efflux pump | tetracycline antibiotic | antibiotic efflux | 99.25 | 100.00 |  |
| Strict | tet(A) |  | protein homolog model | major facilitator superfamily (MFS) antibiotic efflux pump | tetracycline antibiotic | antibiotic efflux | 99.74 | 94.10 |  |
| Strict | APH(6)-Id |  | protein homolog model | APH(6) | aminoglycoside antibiotic | antibiotic inactivation | 99.64 | 100.00 |  |
| Strict | sul2 |  | protein homolog model | sulfonamide resistant sul | sulfonamide antibiotic | antibiotic target replacement | 99.63 | 100.00 |  |
| Strict | APH(3'')-Ib |  | protein homolog model | APH(3'') | aminoglycoside antibiotic | antibiotic inactivation | 99.25 | 100.00 |  |
| Strict | AAC(6')-Iy |  | protein homolog model | AAC(6') | aminoglycoside antibiotic | antibiotic inactivation | 97.93 | 118.62 |  |
| Strict | Escherichia coli EF-Tu mutants conferring resistance to Pulvomycin | R234F | protein variant model | elfamycin resistant EF-Tu | elfamycin antibiotic | antibiotic target alteration | 99.49 | 96.33 |  |
| Strict | tetR |  | protein overexpression model | major facilitator superfamily (MFS) antibiotic efflux pump | tetracycline antibiotic | antibiotic target alteration, antibiotic efflux | 100.0 | 100.00 |  |
| Strict | Escherichia coli soxR with mutation conferring antibiotic resistance |  | protein overexpression model | ATP-binding cassette (ABC) antibiotic efflux pump, major facilitator superfamily (MFS) antibiotic efflux pump, resistance-nodulation-cell division (RND) antibiotic efflux pump | fluoroquinolone antibiotic, cephalosporin, glycylcycline, penam, tetracycline antibiotic, rifamycin antibiotic, phenicol antibiotic, disinfecting agents and antiseptics | antibiotic target alteration, antibiotic efflux | 96.05 | 98.70 |  |
| Strict | Escherichia coli soxS with mutation conferring antibiotic resistance |  | protein overexpression model | ATP-binding cassette (ABC) antibiotic efflux pump, major facilitator superfamily (MFS) antibiotic efflux pump, resistance-nodulation-cell division (RND) antibiotic efflux pump, General Bacterial Porin with reduced permeability to beta-lactams | fluoroquinolone antibiotic, monobactam, carbapenem, cephalosporin, glycylcycline, cephamycin, penam, tetracycline antibiotic, rifamycin antibiotic, phenicol antibiotic, penem, disinfecting agents and antiseptics | antibiotic target alteration, antibiotic efflux, reduced permeability to antibiotic | 94.39 | 100.00 |  |
