# Supplementary material for: Pangenome diversification and resistance gene characterization in Salmonella Typhi prioritized RfaJ as a significant therapeutic marker
Source: J Genet Eng Biotechnol. 2023 Nov 17;21:125. doi: 10.1186/s43141-023-00591-w (PMC10656401; doi:10.1186/s43141-023-00591-w)
Supplement: Supplementary file 4 — Additional file 4: Table S4. Resistance gene identified from the unique genome of 119 S. Typhi. [file 43141_2023_591_MOESM4_ESM.docx]

Supplementary Table 4: Resistance gene identified from the unique genome of 119 S. Typhi

| RGI Criteria | | ARO Term | | | SNP | Detection Criteria | | AMR Gene Family | Drug Class | | Resistance Mechanism | | % Identity of Matching Region | | % Length of Reference Sequence | | |
| --- | --- | --- | --- | --- | --- | --- | --- | --- | --- | --- | --- | --- | --- | --- | --- | --- | --- |
|  |  | |  |  | | |  | | |  | |  | |  | |  |  |
| Perfect | tet(D) | |  | protein homolog model | | | major facilitator superfamily (MFS) antibiotic efflux pump | | | tetracycline antibiotic | | antibiotic efflux | | 100.0 | | 100.00 |  |
| Perfect | SHV-1 | |  | protein homolog model | | | SHV beta-lactamase | | | carbapenem, cephalosporin, penam | | antibiotic inactivation | | 100.0 | | 100.00 |  |
| Perfect | APH(3')-Ia | |  | protein homolog model | | | APH(3') | | | aminoglycoside antibiotic | | antibiotic inactivation | | 100.0 | | 100.00 |  |
| Perfect | OXA-10 | |  | protein homolog model | | | OXA beta-lactamase | | | carbapenem, cephalosporin, penam | | antibiotic inactivation | | 100.0 | | 100.00 |  |
| Perfect | aadA | |  | protein homolog model | | | ANT(3'') | | | aminoglycoside antibiotic | | antibiotic inactivation | | 100.0 | | 100.00 |  |
| Strict | cmlA1 | |  | protein homolog model | | | major facilitator superfamily (MFS) antibiotic efflux pump | | | phenicol antibiotic | | antibiotic efflux | | 99.76 | | 100.00 |  |
